# Supplementary material for: Targeted and Untargeted Metabolomic Analyses Reveal Organ Specificity of Specialized Metabolites in the Model Grass Brachypodium distachyon
Source: Molecules. 2022 Sep 13;27(18):5956. doi: 10.3390/molecules27185956 (PMC9506550; doi:10.3390/molecules27185956)
Supplement: Supplementary file 1 [file molecules-27-05956-s001.zip › Supplementary Figure S1.pdf]

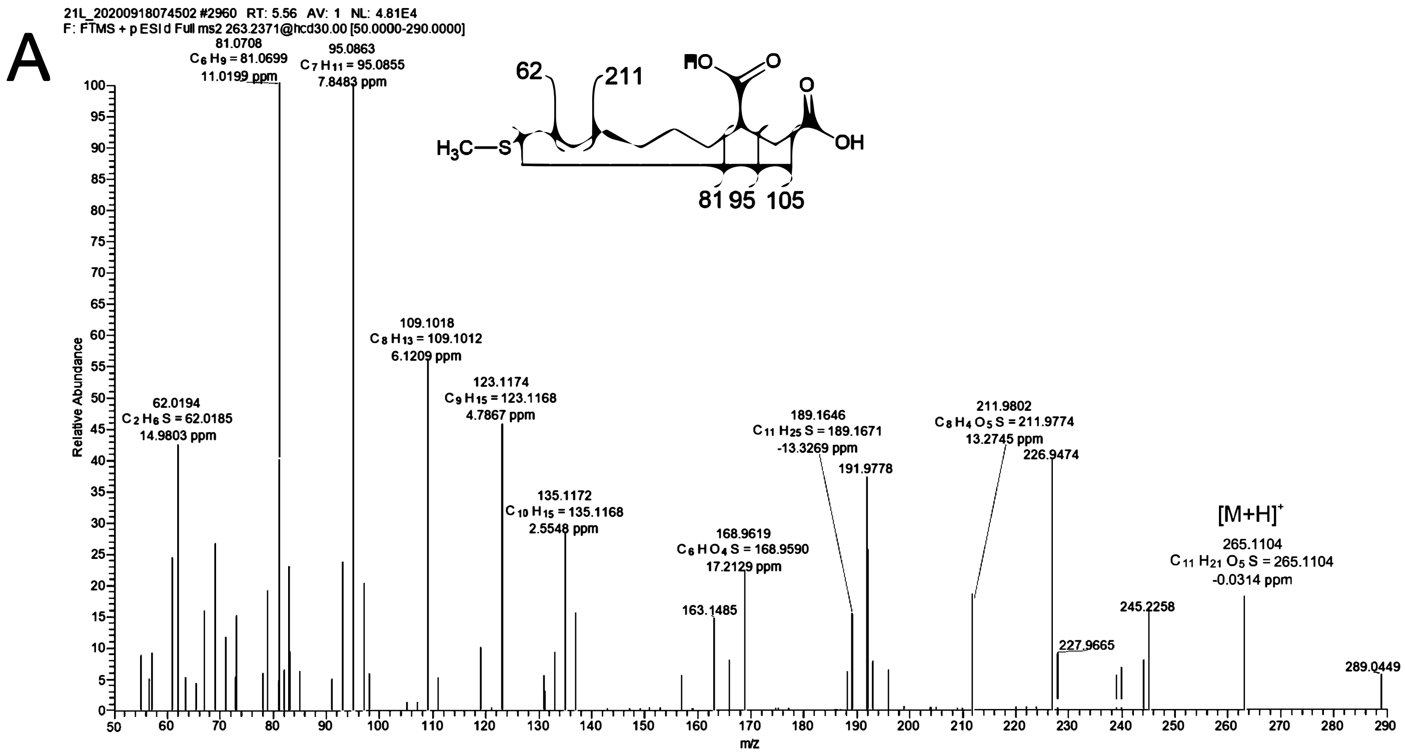

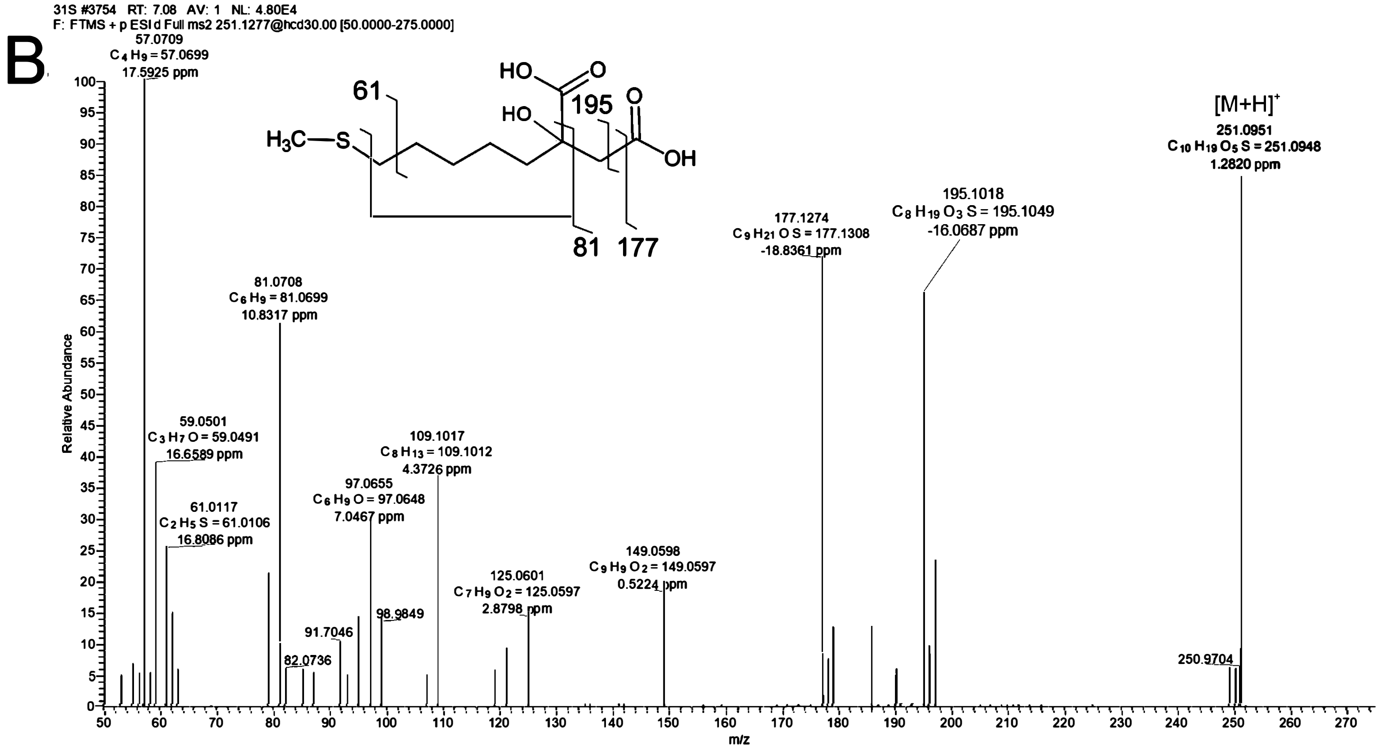

Supplementary Figure S1. High resolution MS/MS spectra obtained in positive ionization of identified isomeric structures of sulphur-containing dicarboxylic acids whose presence in Brachypodium was suggested by pathway enrichment analysis. (A) 6'-methylthiohexylmalic acid; (B) 5'-methylthiopentylmalic acid. Main product ions and calculated chemical formulas have been indicated. Postulated structures with main fragmentation schemes are shown representatively as 2-(6'-methylthio)hexylmalic and 2-(5'-methylthio)pentylmalic acid, however, precise isomer identification requires further studies.
