# Supplementary material for: Targeted and Untargeted Metabolomic Analyses Reveal Organ Specificity of Specialized Metabolites in the Model Grass Brachypodium distachyon
Source: Molecules. 2022 Sep 13;27(18):5956. doi: 10.3390/molecules27185956 (PMC9506550; doi:10.3390/molecules27185956)
Supplement: Supplementary file 1 [file molecules-27-05956-s001.zip › Supplementary Figure S2.pdf]

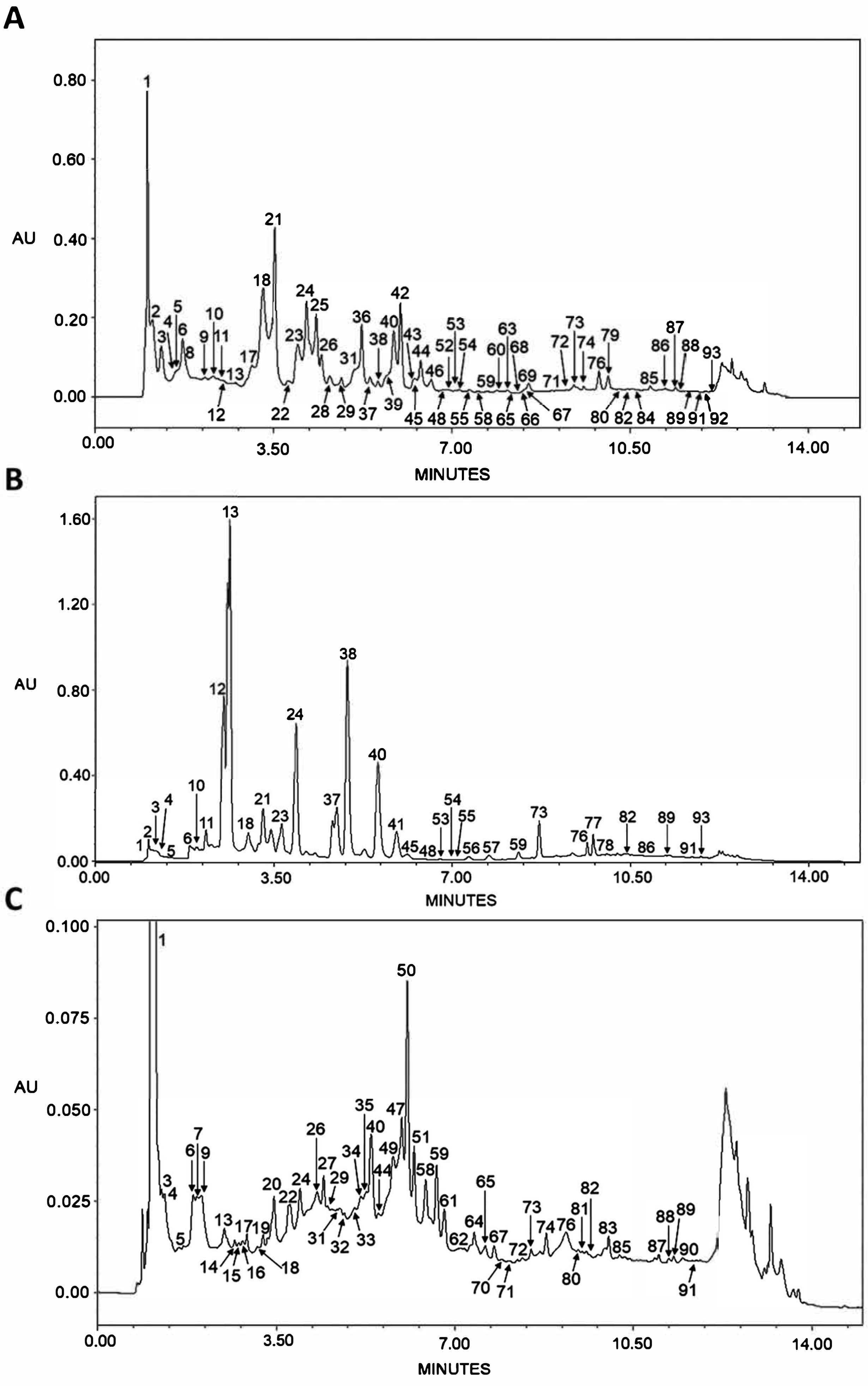

Supplementary Figure S2. Representative UV chromatograms recorded at 270 nm during analysis of samples prepared from (A) leaves, (B) roots, (C) spikes. Numbers indicate peaks of identified metabolites accordingly with Table 3.
